# Deposition of Aluminide Coatings onto AISI 304L Steel for High Temperature Applications

**DOI:** 10.3390/ma15124184

**Published:** 2022-06-13

**Authors:** Zubia Anwer, Muhammad Tufail, Ali Dad Chandio

**Affiliations:** Materials and Surface Engineering Materials Laboratory, Department of Metallurgical Engineering, NED University of Engineering and Technology, Karachi 75270, Pakistan; zubia@neduet.edu.pk (Z.A.); pvc@neduet.edu.pk (M.T.)

**Keywords:** aluminide coatings, electro-deposition, β-NiAl, oxidation and mechanical properties, AISI 304L, α-Al_2_O_3_, hot corrosion

## Abstract

The nickel aluminides are commonly employed as a bond coat material in thermal barrier coating systems for the components of aeroengines operated at very high temperatures. However, their lifetime is limited due to several factors, such as outward diffusion of substrate elements, surface roughness at high temperatures, morphological changes of the oxide layer, etc. For this reason, inter-diffusion migrations were studied in the presence and absence of nickel coating. In addition, a hot corrosion study was also carried out. Thus, on one set of substrates, nickel electrodeposition was carried out, followed by a high activity pack aluminizing process, while another set of substrates were directly aluminized. The microstructural, mechanical, and oxidation properties were examined using different characterization techniques, such as SEM-EDS, optical microscopy, XRD, optical emission spectroscopy, surface roughness (Ra), and adhesion tests. In addition, the variable oxidation temperatures were employed to better understand their influence on the roughness, degree of spallation (DoS), and morphology. The results show that AISI 304L substrates do not respond to aluminizing treatment, i.e., no aluminide coating was formed; rather, a nearly pure aluminum (or alloy) was observed on the substrate. On the contrary, successful formation of an aluminide coating was observed on the nickel-electrodeposited substrates. In particular, a minimum amount of migrations were noted, which is attributed to nickel coating. Moreover, the scratch test at 10 N load revealed neither cracking nor peeling off, thereby indicating good adhesion of the aluminide coating before oxidation. The as-aluminized samples were oxidized between 700 °C to 1100 °C in air for 8 h each. The degree of spallation showed an incremental trend as temperatures increased. Likewise, oxide morphologies showed temperature dependence. On the other hand, average surface roughness (from Ra = 2.3 µm to 5.8 µm) was also increased as temperatures rose. Likewise, the mass gain showed linearity as temperatures increased during oxidation. The hot corrosion responses of electrodeposited-aluminized samples were superior among all specimens. An extensive discussion is presented based on the observations noted above.

## 1. Introduction

A significant number of industrial processes operate in aggressive environments characterized by high temperatures, increased humidity, increased temperature gradients, high pressure, large stresses on individual materials, and the presence of oxidizing and corroding environment, as well as internally formed or externally ingested particulate content, and all cause erosion and impact damage [1]. Stainless steel and its alloys have been commonly used in high-temperature oxidation and hot corrosion environments, and one of the key objectives of research in this area has been to extend their life span. AISI 316L and 304L stainless steels are currently the most popular austenitic stainless steels used in nuclear power plants [2]. Extensive research is being done on the mechanical properties and corrosion activity of AISI 304L at high temperature. Since AISI 304L has a lower carbon content (about 0.03 percent), it produces less hazardous or deleterious carbide precipitation during coatings [3]. It is well understood that a passive film formed on the electrode interface from a mixture of iron and chromium oxides/hydroxide causes reduction in the corrosion rate [4]. In gas turbine engines, the corrosion phenomena also depends on the chemistry of molten salt environment, e.g., chlorides, fluorides, sulphates, nitrates, etc. The high temperature hostile environment can cause the oxide coating to become non-protective. As a consequence, the underlying alloy undergoes severe corrosion and degradation [5].

However, the rapid oxidation may be an important cause of their degradation [6]. In order to protect these alloys from high temperature oxidation, nickel coating is employed on these materials, along with an aluminizing treatment to form protective, thermodynamically stable α-Al_2_O_3_.

The surface is the only component of a substance that must coexist with the external environment; however, the majority of engineering failures start on the surface and then spread to the components, causing fatigue, wear, corrosion, and oxidation. Surface modification can be done to enhance these properties using a variety of technique that include depositing a suitable coating material onto the surface [7]. Coatings for high-temperature components have appealing properties, such as oxidation and hot corrosion resistance, as well as the ability to preserve resilience, cohesion, and other characteristics. At high temperatures, coatings form a compact, adherent oxide scale that provides a diffusion barrier against the transport of reactants between the high-temperature gases and the underlying metal, thus greatly reducing further attack by the atmosphere. Typically, these coatings contain β-NiAl as a principal constituent phase in order to have sufficient aluminum content to form an Al_2_O_3_ scale at elevated temperatures [8]. Different coatings enriched the element, forming a uniform adhering, slow growing, and dense oxide scale, and were applied to the AISI 304L substrate to improve oxidation and corrosion resistance while preserving mechanical properties. Coatings made of diffusion aluminides are examples of such coatings. Adding sub-specific alloying elements will strengthen the protective properties even more [9,10].

Nickel aluminide coatings are known for their high temperature mechanical strength and their ability to withstand corrosion. Forming a protective external alumina scale improves oxidation and hot corrosion resistance [10]. β-NiAl coatings have received a lot of attention because of their combination of desirable properties, including high melting temperature, as well as strong thermal conductivity [11,12]. Nickel-aluminum intermetallic alloys based on NiAl or Ni_3_Al have shown good resistance to high temperature oxidation [12,13]. Some of the common ways by which these coatings can be applied are: thermal spraying, weld overlaying, and pack cementation (aluminizing), sputtering, physical vapor deposition, chemical vapor deposition, etc. [14]

Currently, many researchers have studied the deposition of Ni-aluminides on various alloy steels and mild steel. For this purpose, the nickel electrodeposition, followed by high/low activity pack aluminizing, may be applied on the substrate. The purpose of nickel coating is to provide a diffusion barrier between the aluminized layer and the substrate, thus improving the overall life of the alloy. For the purpose of Ni-electrodeposition, sulfamate baths, or Watts bath, can be used. The properties and microstructures of the electrodeposits are closely related to the electrolyte composition and electroplating parameters [14]; for example, nickel sulfamate baths are widespread in high speed electrodeposition, electroforming, and electro joining processes because the resulting nickel deposits exhibit low internal stress and good ductility [1,15].

At low oxidation temperatures, i.e., less than 1000 °C, the formation of transient alumina phases, such as γ’, θ and 𝛿 Al_2_O_3_, were observed, mostly in case of aluminized alloys. These oxides have an FCC closed pack oxygen lattice with differences in their cation arrangements. These oxides show high aluminum diffusion, and thus grow faster at the early stages of oxidation. They have a whiskers morphology, due to their unique transport mechanism. At the epitaxy of NiAl and FeAl, the nucleation of γ’, 𝛿 Al_2_O_3_ is highly favored [16,17]. The transformation sequence is γ’, θ and α-Al_2_O_3_ , as studied in most cases, but in some studies, direct formation of thermodynamically stable α-Al_2_O_3_ is possible at high temperatures [18]. Since the growth rate of metastable Al_2_O_3_ phases is faster than α-Al_2_O_3_, they are undesirable as protective oxides [19]. 

Furthermore, Y. Zhang et al. determined the environmental and oxidation resistance of aluminide coatings deposited onto Fe-9Cr-1Mo (ferritic) and 304L (austenitic) stainless steels. The authors concluded that the coating spalled due to oxidation at high temperature (700 °C); however, no corrosion damage was observed when exposed to air+10 Vol % H_2_O environments [20]. Similarly, in another study on β-NiAl coatings deposited onto AISI 304 L samples, the corrosion resistance was reported to be improved over that of bare metal [21].

In this work, two sets of samples were prepared: (1) AISI 304L alloy was directly aluminized and (2) nickel electroplating was carried out on the same alloy, and this was followed by pack aluminizing and diffusion annealing treatments. The very aim of this study was to better understand the response of alloys towards aluminizing and the influence of temperature on oxidation events and the hot corrosion response of the resultant coatings. In addition, the effect of nickel plating was assessed in terms of coating formation and interdiffusion migrations. 

## 2. Experimental Approach

### 2.1. Materials

The AISI 304L steel was used in this study, the nominal composition of which is shown in Table 1. The microstructure of the alloy consists of austenite grains with annealing twins [22]. The substrates of 10 × 10 × 2 mm^3^ were cut using Micracut 201 into small discs, ground progressively up to 1000 grit emery papers, and thereafter polished and cleaned ultrasonically in an ethanol bath.

### 2.2. Methods

#### 2.2.1. Nickel Electrodeposition

For nickel coating, the nickel and sulphate containing Watts bath [23] was used, with platinum mesh as the anode of the electrodeposition setup.

Before electrodeposition, the surfaces of the substrates were cleaned in ethanol and sulfuric acid for activation. After activation, the samples were immediately set in a deposition bath as a cathode.

The Watts bath composition and deposition parameters are summarized in Table 2 and Table 3.

Nickel plating is the same as other electrodeposition techniques that utilize soluble metal anodes: the means direct current is allow to flow between the anode and the cathode that are immersed in an aqueous, conductive solution of nickel salts. The passage of electric current in the conductive medium allows the coating of nickel on the cathode and the consumption of the anode. The nickel is present as divalent (Ni^2+^) in the solution. The positively charged ions Ni^2+^ react with two electrons to form metallic nickel (Ni^0^) at the cathode surface. Simultaneously, the reverse process is taking place at anode, where the anode has been dissolved in the solution as Ni^2+^ [24].

#### 2.2.2. Pack Aluminizing

A powder mixture of 30 g by weight was used for high activity aluminizing. The mixture contained 24.9 g alumina (Al_2_O_3_), 4.5 g of aluminium (Al), and 0.6 g of ammonium chloride (NH_4_Cl) [25]. At first, the mixture was dried in an oven at 80 °C for 1 h to remove moisture and contaminants. Using a heat treatment furnace with an argon-controlled atmosphere, the samples were aluminized at 900 °C for 3 h in a sealed zirconia crucible. The samples were allowed to cool in the furnace after the required time interval. The samples were ultrasonically cleaned after the aluminizing process.

#### 2.2.3. Diffusion Annealing

After electrodeposition, the samples were subsequently subjected to the diffusion annealing process in a tube furnace to form a protective layer of β-NiAl. The samples were heated to a temperature of 1000 °C with a heating rate of 10 °C/min in an argon gas environment for 120 min.

**Figure 1:**
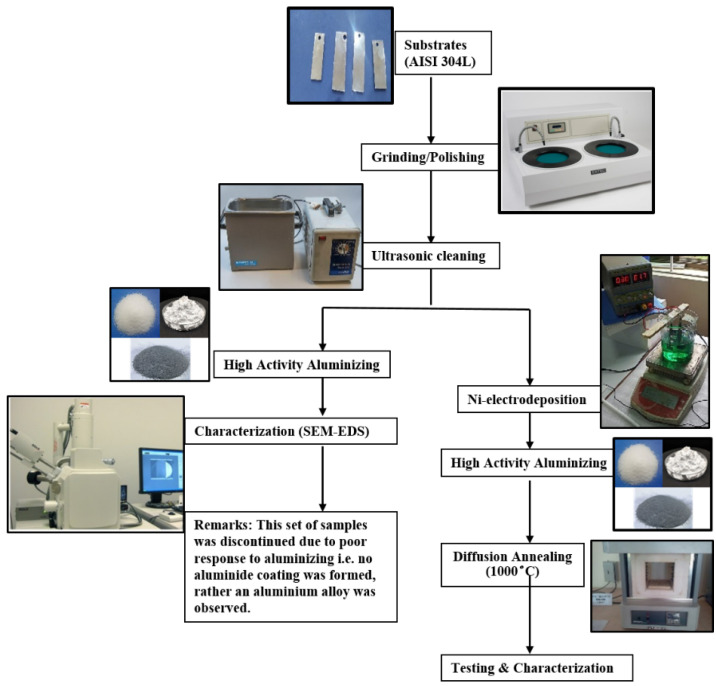
The process flow chart for two sets of substrates.

Moreover, the summary of the overall process for the coating is shown in the process flow diagram (Figure 1).

#### 2.2.4. Oxidation

After coating and the subsequent diffusion annealing treatment, the samples were isothermally oxidized at 700 °C to 1100 °C for 8 h. The mass changes were recorded before and after oxidation using a precision weight balance (TX-200, Akira, Japan). Thereafter, optical microscopy and X-ray diffraction techniques were utilized to analyze the oxide/substrate phases/morphologies.

#### 2.2.5. Etching

To perform optical microscopy of the coated samples before oxidation, a solution was prepared, i.e., 45% CH_3_COOH + 35% HNO_3_ + 10% HCL + 10%H_3_PO_4_ for etching purpose.

#### 2.2.6. Corrosion Testing

The hot corrosion test [5,26] of the bare, as-aluminized, and electrodeposited-aluminized samples was performed in a muffle furnace in static air. A mixture of Na_2_SO_4_: NaCl (3:1 by weight) was used as the corrosive medium. The salt mixture was applied (coated) in the quantity of 1 mg/cm^2^ onto the alloyed samples. The samples were weighed before and after application of the salt. Thereafter, the samples were loaded in the alumina crucible and placed in furnace for 8 h in the temperature range of 700 °C–1100 °C. After that, the samples were cooled in static air and then removed; this was followed by washing in an ultrasonic bath for 15 min in order to remove remaining salt. After drying, the samples were allowed to dry and weighed again using a precision electronic balance.

### 2.3. Testing and Characterization

Scanning electron microscopy (TESCAN, Series Vega 3 XMU Model 116-0037, Brno, Czech Republic) with EDS (BRUKER Model#1119-0000-400, Billerica, MA, USA) and optical microscopy (Metkon, IMM-901, Bursa, Turkey) were used for microscopic analysis. The energy dispersive spectroscopy was used for chemical analysis. The phase analysis was done with the help of the X-ray diffraction technique (PAN analytical X’Pert Pro XRD DY3313, Amsterdam, The Netherland). The scratch test was conducted to examine the adhesion of as-coated samples using scratch tester (ST-30, Teer coatings.co, Droitwich, UK). In addition, the roughness test was also carried out using surface roughness tester (KR-310, Beijing, China).

## 3. Results and Discussion

### 3.1. Microstructures

**Figure 2:**
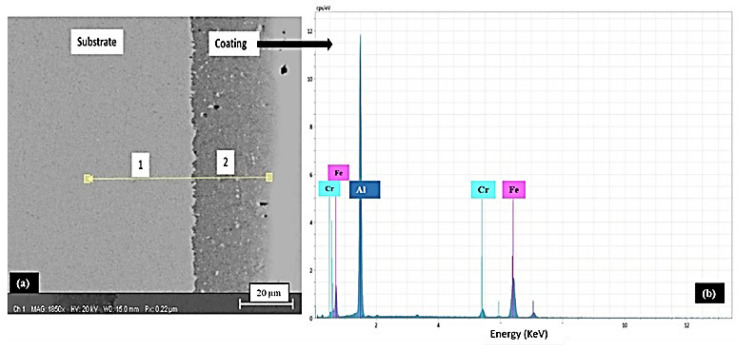
(**a**) The SEM micrograph of the AISI 304L substrate after the aluminizing treatment and (**b**) the EDS analysis of the same.

Figure 2a shows the cross-section image of the as-aluminized sample, without nickel deposition, whereas 2b shows the EDS analysis of the as-aluminized layer. Table 4 shows the chemical composition of the substrate and the as-aluminized layer after pack aluminizing.

With reference to the phase diagram of the Fe-Al alloy system, aluminizing above 1000 °C could form various phases, such as FeAl_3_, FeAl_2_, or FeAl, etc., in addition to solid solutions of Fe and Al [27], as per knowledge of the Al-Fe phase diagram and the data provided that an aluminium rich intermetallic phase (Al_13_Fe_4_) can be present with a very limited dilution of Fe [28]. Thus, aluminides of iron will not be formed in this case of AISI 304 L after pack aluminizing, as shown in Table 4 and Figure 2.

**Figure 3:**
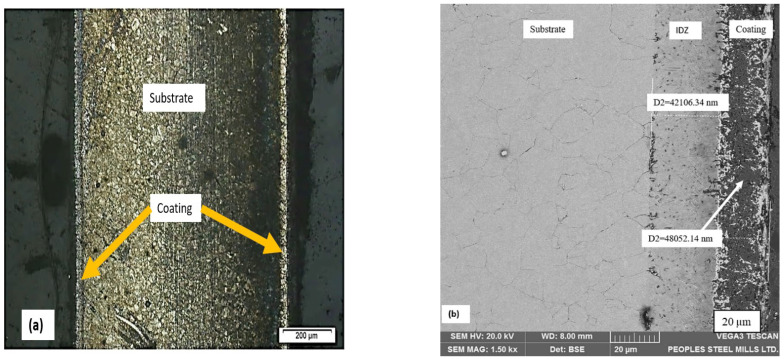
Cross-sections of AISI 304L coated substrates exhibiting coating layer by (**a**) cross-section taken by optical microscopy at 50X and (**b**) the SEM micrograph, which exhibits the outer layer and interdiffusion zone that are characteristic features of typical aluminide coating.

Figure 3a,b shows the cross-sectional image of aluminide coating in its as-deposited condition after annealing. It appears that a typical aluminide layer is formed, having both an interdiffusion zone (IDZ) and an outer layer. In addition, Table 5 shows the chemical composition of the βNiAl. The aluminide formed here contains the chromium and iron entrapment, due to outward diffusion of the substrate.

The microstructure from a cross-section of aluminized (at 900 °C for 3 h) sample on pre-electroplated AISI 304L is presented in Figure 3a. The microstructure reveals that the aluminized sample has a bilayer structure. The main coating zone and an interdiffusion zone are shown in Figure 3b. The coating zone thickness was measured to be around 48 µm, whereas the IDZ has a thickness of 42 µm.

**Figure 4:**
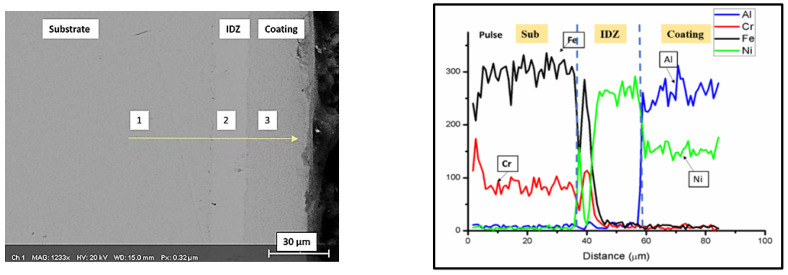
The SEM micrograph of the coating cross-section (**left**) along with corresponding line scan of the same (**right**).

**Figure 5:**
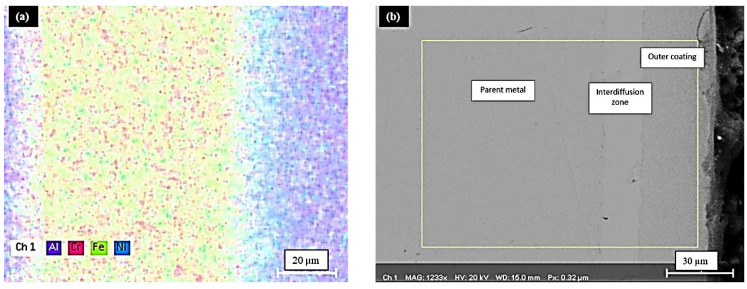

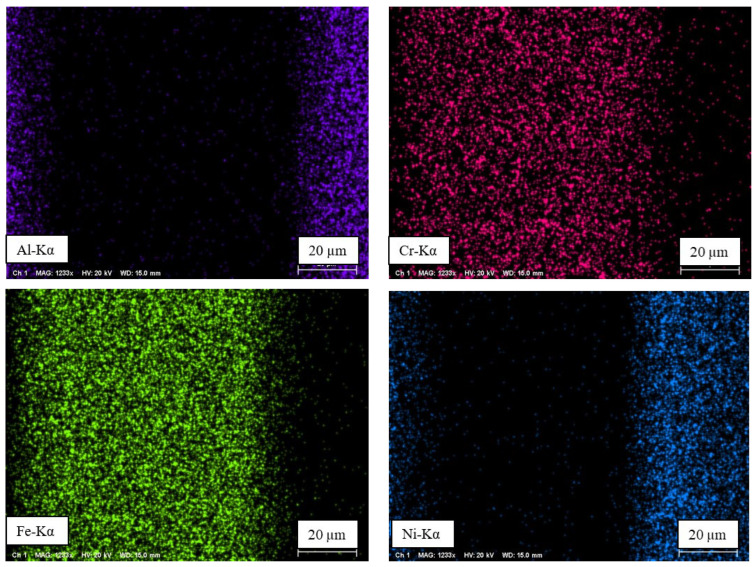
(**a**) The elemental mapping of the coating cross-section, exhibiting the typical aluminide layer (**b**) The mapped area.

The distribution of the elemental species of Ni, Al, Cr, and Fe throughout the entire thickness of the aluminized sample is shown Figure 4b, whereas their elemental distribution is shown in Figure 5a,b.

With reference to the binary phase diagram of the Ni-Al system [29,30,31], there is the possibility of five types of Ni-Al intermetallic compounds, i.e., NiAl_3_, Ni_3_Al, NiAl, Ni_2_Al_3_, and Ni_5_Al_3_. Among these intermetallic compounds, only two are stable phases at high temperature, i.e., Ni_3_Al and NiAl. To stabilize Ni_3_Al, a large amount of Ni outer-diffusion is required from the substrate. The stoichiometry of β-NiAl has an effect on the expected composition of the oxide layer. In case of Al-rich β-NiAl, as shown in Table 5, the aluminum diffusion is expected to dominate during oxidation [32].

### 3.2. Phase Analysis

**Figure 6:**
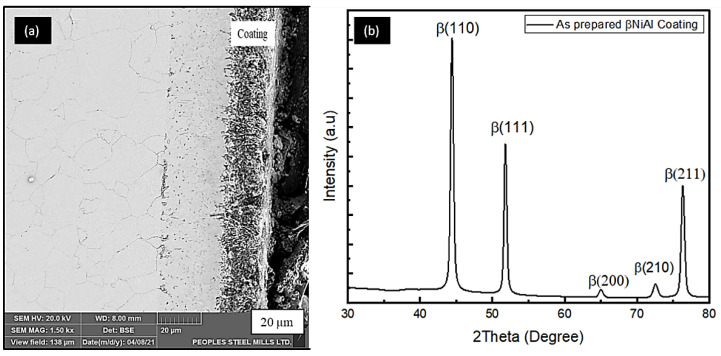
The XRD spectrum of the coating formed after aluminizing (**a**) as deposited on the substrate, (**b**) XRD analysis for the same. Typical peaks could be seen in the spectrum. Thus, this translates that the successful coating procedures were adopted.

Figure 6 showed the presence of β-NiAl structure as being the major phase of the coating formed after the aluminizing treatment. Similar XRD results are reported in the work done by Chandio et al. and Brumm et al. [33,34].

### 3.3. Adhesion Test

**Figure 7:**
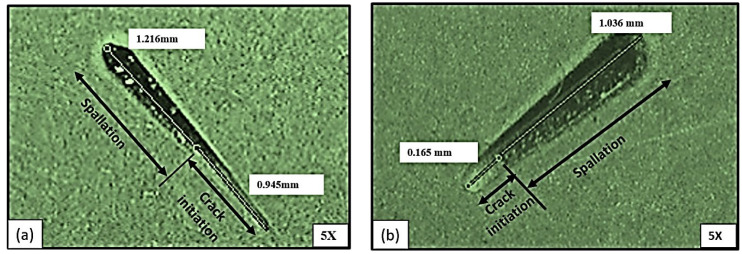
Scratch mark observed during the adhesion test at 5X on (**a**) electrodeposited aluminized steel, (**b**) without electrodeposition aluminized steel.

To study the adhesion of the coating, the scratch test [35] was performed using the diamond indenter of the machine. The residual scratch was monitored by using stereoscopy, as shown in Figure 7. The low magnification of 5X was used to measure the crack distance. After calculation, the critical load of thin film was found at 20 N, which is the maximum load of the testing equipment. In Figure 7a, the absence of cracks and the distance covered by electrodeposited aluminide steel indicates good adhesion, since the crack initiation distance is greater than that without the electrodeposited aluminized counterpart. It is clear in Figure 7b that exhibits rapid crack initiation and fast spallation, as compared to sample shown in Figure 7a.

### 3.4. Isothermal Oxidation Test

#### 3.4.1. Weight Gain and Oxide Morphologies

**Figure 8:**
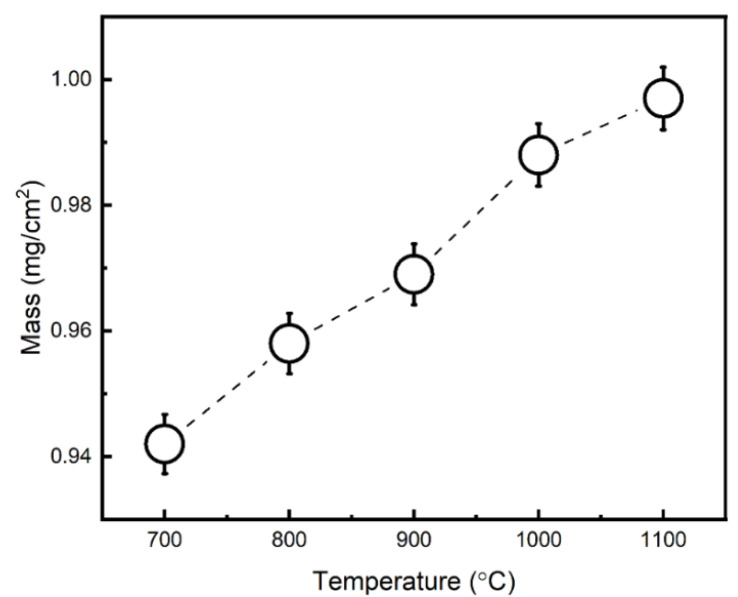
Mass gain with respect to different exposure temperatures, i.e., 700 °C–1100 °C.

The mass change of AISI 304 L after exposure at various temperatures, i.e., 700 °C–1100 °C for a period of 8 h, is shown in Figure 8. The temperature change exhibited a significant effect on the mass gain plot, i.e., an incremental trend was noticed upon temperature rise. This is attributed to the formation of the alumina phases, i.e., transient oxides to stable α-Al_2_O_3_ at high temperatures.

**Figure 9:**
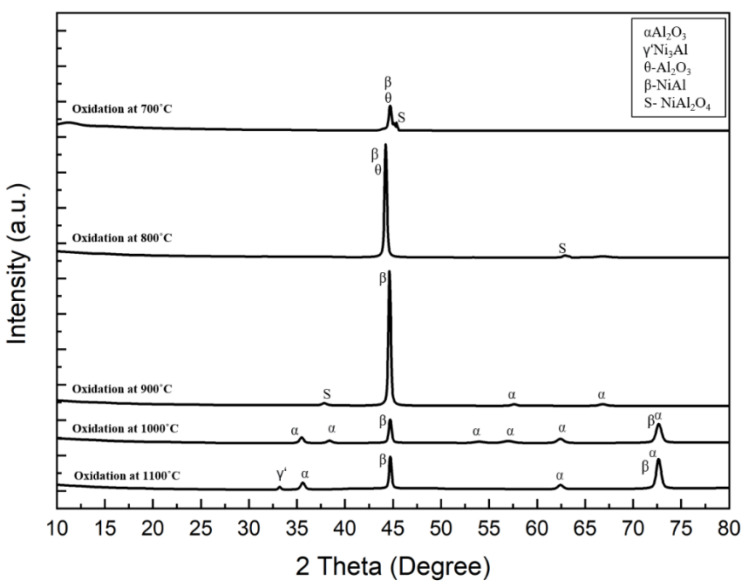
The XRD spectrum of aluminized samples after oxidation treatment at 700 °C–1100 °C for 8 h. The peaks confirm the presence of various oxide morphologies for each oxidizing temperature.

There is a high probability for the formation of more than one oxide during oxidation, depending on the thermodynamics of chemical reactions and certain parameters such as, temperature, oxygen partial pressure, and chemical composition [36,37,38]. At the beginning of the oxidation reactions, the transient aluminium oxides are nucleated at the surface of the material. Since the nucleation rate of various oxides is different, according to some studies, the nucleation rate of oxides is a function of the initial concentration of the elements present at the interface between the oxygen environment and the as-aluminized layer [38]. The high concentration of aluminium at the coating surface before oxidation reaction suggests the formation of corundum- and aluminium-based spinel products in the temperatures range of 700 °C to 1100 °C, as shown in Figure 9. As the exposure temperatures increases, there will be an equilibrium maintained between the inward oxygen diffusion and outer Al diffusion [39].

Figure 9 represent the X-ray diffraction pattern of the oxidized samples. The XRD was operated at 30 mA, 40 kV using CuKα radiations. At low temperatures, 700 °C–800 °C, transient oxides, i.e., θ-Al_2_O_3_, along with the spinel phase of NiAl_2_O_4_ [35], were formed, thereby triggering weight gains, as indicated earlier.

However, at 900 °C, the θ-Al_2_O_3_ changed into α-Al_2_O_3_ [39,40,41]. The PBR (Pilling–Bedworth ratio) for α-Al_2_O_3_ formation is greater than 1, thus providing good protection against high temperature oxidation [42]. However, spinel phases were also formed, as noted in earlier Figure 9. Moreover, when the temperature was increased between 1000 °C and 1100 °C, the α-Al_2_O_3_ was the only major oxide formed.

Nevertheless, a peak of Ni_3_Al were found, as shown in the XRD spectrum. It should be noted that the peaks of aluminide are from the coatings, since some oxides were found to be damaged/spalled. In addition, the oxidation time was not very high; therefore, β-NiAl signals were seen in the X-ray diffraction spectra.

#### 3.4.2. Degree of Spallation (DoS)

**Figure 10:**
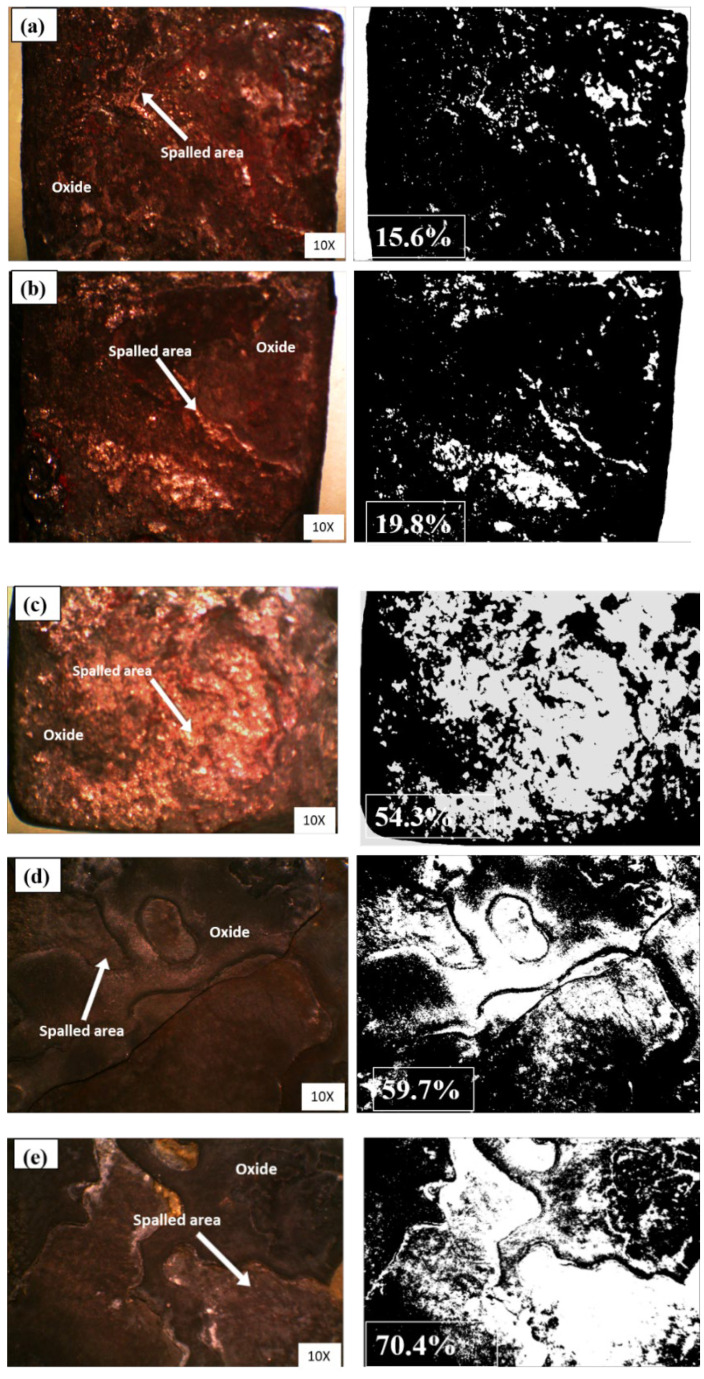
The oxide morphologies using a stereoscope at 50X (**a**) at 700 °C, (**b**) 800 °C, (**c**) 900 °C, (**d**) 1000 °C, and (**e**) 1100 °C. The left column of the images indicates the real-time oxides captured using stereo microscopy, while the right side shows the phase fraction images taken to understand the spallation resistance of the samples.

Immediately after oxidation, the oxidized samples were analysed under low magnification of 10X using stereo microscopy. The surface measurements of spalled (damaged) and unspalled (undamaged) areas were assessed to estimate the DoS. Figure 10a–e shows the oxides taken into account for the DoS assessments. The degree of spallation was taken as a function of the oxidation temperatures.

**Figure 11:**
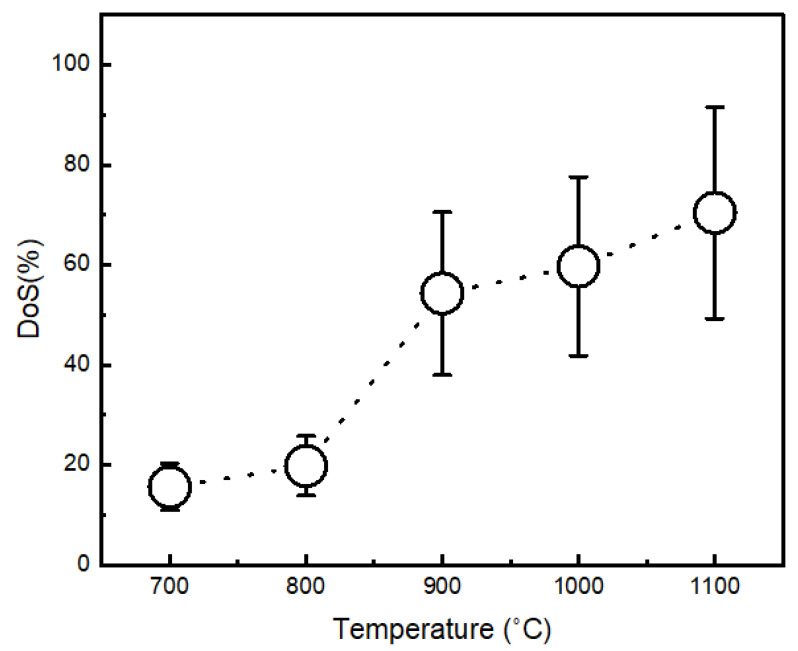
The degree of spallation (DoS) of the samples. The DoS was measured using spalled and unspalled areas. The time for study is the same, i.e., 8 h.

After considering all oxidized samples, it appeared that the spallation is more severe for the samples oxidized at higher temperatures, i.e., 1000 °C and 1100 °C, as shown in Figure 11. This was attributed to poor adhesion.

At high temperatures, the thermal stresses can induce an oxide layer due to difference in the coefficient of thermal expansion, or contraction between the exposed surface and the oxide [38]. Usually, the scale formed under compressive stresses upon cooling results in the spallation of oxides [43].

### 3.5. Surface Roughness (Ra)

The surface roughness tester (KR310) was used to find out the values of R_a_ (arithmetic mean deviation of profile), R_z_ (maximum height of profile), and R_max_ (total peak to valley height) before and after oxidation treatment. The vertical displacement was recorded for the lateral movement of the sensor. The estimated values of the average roughness are presented in Table 6. The cut-off length of 0.80 mm was used.

The surface roughness of the initial sample could have a beneficial effect on the oxidation mechanism. Since it has been reported that upon the oxidation of stainless steels, a rougher surface induces more surface defects, thereby increasing the chances of protective oxide formation [44,45].

It can be seen here that the surface roughness of the oxidized samples at 700 °C and 1100 °C has been increased from 2.31 µm to 5.82 µm, respectively, as compare to the average roughness of the received sample (1.234 µm) before oxidation treatment.

The increase in surface roughness is due to oxidation at high temperatures, thereby leading the rumpling phenomenon, which is very common and observed elsewhere. The rumpling is due to the growth stress relaxation phenomena, in which the bond coat at high temperatures undergoes plastic deformation due to the compressive stresses in the oxide scale in the direction parallel to the growth stress. Upon cooling from high temperatures, due to thermal expansion mismatch between substrate and the surface oxides, which enhances the stress, the probability of spallation increases [46].

### 3.6. Hot Corrosion

**Figure 12:**
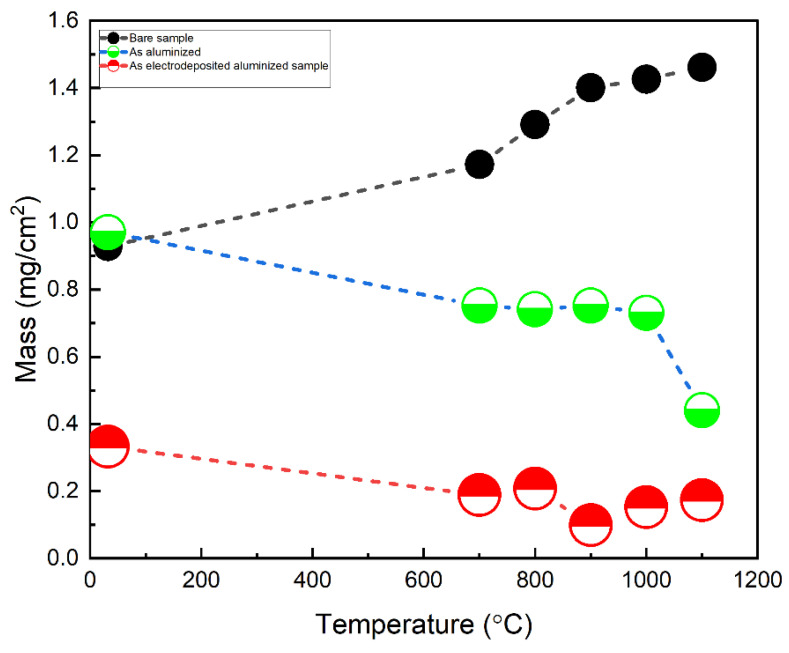
Mass change curves in the temperature range of 700 °C–1100 °C: (i) bare alloy, (ii) as-aluminized, and (iii) electrodeposited-aluminized samples. The environment used is the mixture of Na_2_SO_4_ + NaCl (3:1 by weight).

Figure 12 shows the mass change curves for: bare alloy, as-aluminized, and as electrodeposited-aluminized samples that have been tested in corrosion environment of Na_2_SO_4_ + NaCl for 8 h in the temperature range of 700 °C–1100 °C. It appears very clear from the plot that the bare metals weight gain is incremental upon temperature increase. The highest mass gain could be detrimental, due to formation of the stresses and eventual breakage at long term exposure [5,47].

While the aluminized sample shows more or less similar weight change from 700 °C–1000 °C, when the temperature increased to 1100 °C, a sudden drop in weight was observed. This is attributed to the breakage/spallation of the protective layer. On the contrary, the lowest mass gain was observed at all temperatures for the as electrodeposited-aluminized sample, showing a more or less similar weight change at all temperatures.

## 4. Conclusions

In this study, nickel aluminide coatings were deposited onto AISI 304L substrates. Two sets of coatings were prepared, i.e., with and without the electrodeposition of Ni. The following are the concluding remarks based on the present set of experimental conditions.

When substrates were subjected to aluminizing treatment without Ni electroplating, no aluminide layer was deposited; rather, an aluminium alloy coating was formed.

On the contrary, the nickel electrodeposited samples formed a characteristic β-NiAl layer, exhibiting excellent adhesion. Interestingly, the nickel coating inhibited substrate elements into the coating, i.e., low inter-diffusion was observed.

Oxidation of β-NiAl coating between 700 °C–1100 °C for 8 h was carried out in still air to better understand roughness, morphology, and DoS. After oxidation, θ-Al_2_O_3_ and NiAl_2_O_4_ was formed at a low temperature (700 °C–800 °C). While at 900 °C, the θ-Al_2_O_3_ disappeared, while the spinel phase (NiAl_2_O_4_) remained, in addition to α-Al_2_O_3._ When the temperature was raised between 1000 °C–1100 °C, all transient oxides converted into stable α-Al_2_O_3_. However, at 1100 °C, the β-NiAl–γ’-Ni_3_Al conversion was noticed.

Surface roughness was determined that showed temperature dependence, i.e., the highest values were noted at 1100 °C. Likewise, poor oxidation resistance was also observed at 1100 °C. This is in terms of the degree of spallation. This suggests the incorporation of reactive/noble elements into aluminide for improved DoS.

Moreover, hot corrosion resistance of electrodeposited-aluminized samples was enhanced among all, due to the formation of protective oxides at all temperatures.

## Figures and Tables

**Table 1:** The chemical composition of AISI 304 L alloy.

Substrate	Elemental Composition (Wt. %)									
AISI304L	Fe 71.46	C 0.03	Si 0.4	Mn 1.045	Cr 18.55	Ni 8.003	Mo 0.072	Cu 0.065	S 0.001	P 0.031

**Table 2:** The sulphate bath composition for nickel electrodeposition.

S#	Components	Concentration	Purpose
1.	Nickel sulphate	15 g/100 mL	Ni source
2.	Nickel chloride	6 g/100 mL	Anode activator
3.	Boric acid	3.75 g/100 mL	Buffer source

**Table 3:** The electrodeposition parameters for nickel electrodeposition.

S#	Operating Conditions	Operating Parameter
1.	Temperature	57 °C
2.	Agitation	100 rpm
3.	pH	3.5 ± 1
4.	Time	1 h
5.	Current	75 mA

**Table 4:** The EDS analysis of the AISI 304L samples after aluminizing (point area 2) in comparison to the substrate (point area 1). Please refer to Figure 2a for point area details.

Area Point	Elemental Composition (at. %)				
	Fe	Al	Cr	Ni	Mn
1.	68.60	--	17.79	7.68	4.02
2.	1.32	98.39	0.29	--	--

**Table 5:** The EDS analysis of aluminized samples formed onto AISI 304L samples after nickel electrodeposition. Please note that point 1 is the chemical composition of substrate, while point 2 indicates the interdiffusion zone (IDZ), and the outer layer is shown in point 3. Please refer to Figure 4 for point area details.

Area Point	Elemental Composition (at. %)					
	Fe	Al	Ni	Cr	Mn	C
1.	68.60	--	7.68	17.79	4.02	1.91
2.	22.26	3.54	58.23	15.97	--	--
3.	0.21	52.11	46.85	0.83	--	--

**Table 6:** Roughness values before oxidation and after oxidation.

S #	Conditions	R_a_ (µm)	R_z_ (µm)	R_max_ (µm)
1.	Before oxidation	1.23	8.18	14.78
2.	Oxidation at 700 °C	2.31	14.25	26.51
3.	Oxidation at 800 °C	3.65	17.89	29.04
4.	Oxidation at 900 °C	3.97	24.44	33.70
5.	Oxidation at 1000 °C	6.16	36.39	84.55
6.	Oxidation at 1100 °C	5.82	42.47	87.29

## Data Availability

Not applicable.
